# Combined Effect of CuO Nanoparticles and Lighting on the Growth and Antioxidant Potential of Lettuce in CEA

**DOI:** 10.3390/plants15101477

**Published:** 2026-05-12

**Authors:** Aušra Brazaitytė, Vitalis Laužikas, Justinas Raginskis, Rūta Sutulienė

**Affiliations:** Institute of Horticulture, Lithuanian Research Centre for Agriculture and Forestry, Kauno Str. 30, LT-54333 Kaunas, Lithuania; vitkius1987@gmail.com (V.L.); justinas.raginskis@lammc.lt (J.R.); ruta.sutuliene@lammc.lt (R.S.)

**Keywords:** copper oxide nanoparticle, Cu accumulation, foliar application, hazard quotients, lettuce, lighting, LED, DLI, mineral nutrients, NPs, risk assessment

## Abstract

Nanoparticles (NPs) are becoming more commonly used in agricultural practices for cultivating plants under Controlled Environment Agriculture (CEA). The foliar application of copper oxide (CuO) NPs can enhance the production of bioactive compounds in lettuce without adversely affecting yield. However, there is a lack of data regarding the effects of NPs on plants under various lighting conditions, which is a crucial aspect of CEA. The study aims to find out how different lighting conditions can lead to Cu accumulation, to determine the effects of CuO NPs on lettuce growth, antioxidant potential and mineral elements, and to investigate the potential risk of these NPs’ uptake to human health. Plants were grown in Ebb-type hydroponic systems with red-blue and white-red-blue LED lighting at daily light integral 8.64 and 14.4, sprayed with aqueous suspensions of CuO NPs (40 nm, 30 ppm). The influence was determined on lettuce growth, the enzymatic (GR, APX, CAT, SOD, MDHAR, DHAR) and non-enzymatic (TPC, DPPH, ABTS, FRAP) antioxidants, mineral elements and hazard quotients. Our study showed the synergistic effect of foliar application of CuO NPs and lighting on lettuce. We found that CuO NPs positively influenced lettuce growth and stimulated the antioxidant system, particularly the non-enzymatic components such as phenols, carotenoids, and total antioxidant capacity. This effect was enhanced under a broader wavelength range of white-red-blue light and with a higher daily light integral value of 14.4. The application of CuO NPs significantly increased the Cu content in lettuce. Importantly, the concentration of the used CuO NPs did not reach the limit of Cu ions dangerous to humans, as the calculated intake level remained below safe limits, but it is not determined how much of them remained in the form of NPs.

## 1. Introduction

In recent years, there has been a growing interest in healthy and plant-based diets, leading to a rise in the consumption of vegetables, especially leafy greens, which are rich in bioactive compounds, dietary fibres, minerals, and vitamins [1,2,3]. Lettuce is one of the main leafy vegetables cultivated in Controlled Environment Agriculture (CEA). It has a high growth rate, a short growth cycle, and can be planted at high densities. Research indicates that manipulating various environmental factors can enhance lettuce development and quality cultivated in CEA [2,4,5]. Light is one of the most important environmental factors, essential for photosynthesis and other physiological processes that influence plant growth, development, and phytochemical content. Its characteristics, such as intensity, spectral distribution, and photoperiod, can now be easily controlled using LEDs for lettuce growth [4,5,6]. Boros and co-authors [5] summarised numerous research articles in their review and proposed optimal lighting conditions for indoor lettuce cultivation, accounting for different lettuce varieties and other environmental factors. They showed that for effective lettuce production, the preferred light intensity is 100–300 µmol m^2^ s^−1^, a daily light integral (DLI) of 12–17 mol m^2^ per day, the photoperiod should range from 10 to 18 h per day, and the light spectrum includes red and blue LEDs, which may be supplemented with white, far-red, UV-A, and/or UV-B light.

In addition to environmental factors, various agronomic practices, such as pre-harvest nitrogen limitation, continuous lighting, short-term exposure to low temperatures, high light intensity, etc., are important factors influencing the growth and quality of leafy vegetables [2,7]. Applying nanoparticles (NPs) for pre-harvest spraying could be an effective agronomic practice for lettuce grown in CEA. Numerous studies have demonstrated that copper oxide (CuO) NPs can have both positive and negative effects on lettuce growth in CEA. Moreover, examining NPs in plants under varying lighting conditions is essential, as light acts as a catalyst that influences the reactivity, uptake, and toxicity of CuO NPs [8]. The foliar application of CuO NPs can enhance the production of bioactive compounds in lettuce without adversely affecting yield [9,10]. Specifically, lower concentrations of CuO NPs (≤20 μg mL^−1^) have been found to promote the growth of lettuce seedlings [11]. At higher concentrations, CuO NPs can cause phytotoxicity, leading to reduced biomass, decreased photosynthetic activity, and tissue necrosis in lettuce [12,13]. However, there is insufficient data on the effects of CuO NPs and other NPs on plants under varying lighting conditions. Studies with ice plant have shown different effects of foliar-applied CuO and ZnO NPs under HPS and LED lighting on growth and mineral nutrient content [14]. Zia and co-authors [8] evaluated the toxicity levels of CuO NPs to *Brassica nigra* in vitro under five spectral light conditions (white, blue, red, yellow, and green). Other data showed that the toxicity of ZnO NPs to lettuce seedlings varied under dark or light-dark cycle conditions [15]. Gul and co-authors found that the toxicity of CdO NPs depends on the wavelength of light, which influences the morphological, biochemical, and antioxidant responses of plants [16]. Vatankhah and co-authors [17] discovered that applying TiO_2_ NPs enhances plant growth and productivity, especially under limited light conditions. The analysis of the existing literature suggests that NPs affect plants differently under different lighting conditions, underscoring the importance of further studies in this area. It is hypothesised that WRB LED light, by providing a broader wavelength range, will amplify foliar CuO NPs uptake and stimulate secondary metabolite biosynthesis to a greater extent than RB light, without exceeding permissible Cu intake thresholds. The study aims to determine Cu accumulation in lettuce treated with CuO NPs, to examine how different lighting conditions affect Cu accumulation, to assess the effects of CuO NPs on lettuce growth, antioxidant potential, and mineral elements, and to investigate the potential risk of these NPs’ uptake to human health.

## 2. Results

**Figure 1:**
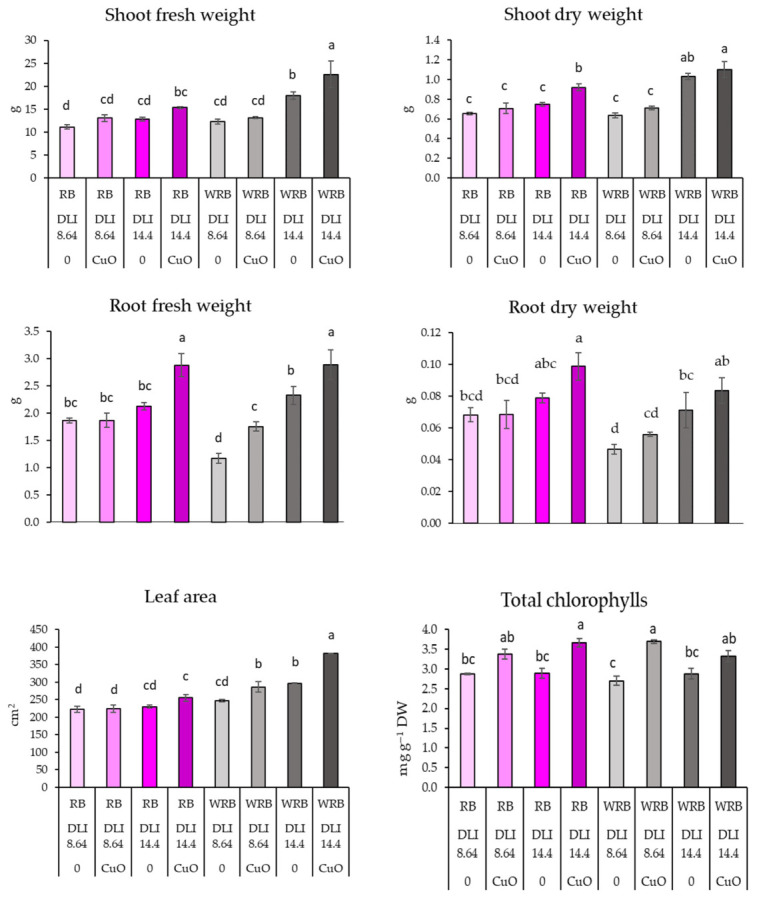
Effects of different lighting and CuO NPs on growth parameters and total chlorophyll content of lettuce. Different letters represent significant differences. RB—red-blue LEDs lamps, WRB—white-red-blue LEDs lamps, DLI—daily light integral, 0—means plants treated with deionised water, CuO—sprayed with CuO NPs, DW—dry weight. Means with different letters within each column are significantly different at the *p* ≤ 0.05 level according to Tukey’s honestly significant difference test.

The results demonstrated a general trend indicating that CuO NPs positively affected lettuce growth (Figure 1; Table 1). However, the significance of this effect was dependent on the illumination conditions. Under the white-red-blue (WRB) LED lighting at a daily light integral (DLI) of 14.4, the shoot fresh weight and leaf area of lettuce increased significantly—by up to 25% and 28%, respectively—when compared to plants that were not treated with CuO NPs. Additionally, CuO NPs resulted in the greatest increase in shoot dry weight, reaching 28% under red-blue (RB) LED light at a DLI of 14.4. Nevertheless, plants were generally greater under WRB at a DLI of 14.4, regardless of the CuO NP treatment. Under a DLI of 14.4, CuO NPs also increased root fresh weight in lettuce grown under both LED light spectra. Spraying CuO NPs under a broader spectrum (WRB) with a lower DLI of 8.64 resulted in nearly a 50% increase in root weight compared to unsprayed plants. Similar trends were observed for root dry weight; however, no significant differences were observed between plants sprayed with CuO NPs and those that were not sprayed.

The results for total chlorophyll content showed a general trend: CuO NPs increased chlorophyll content in lettuce across all lighting conditions. However, significant differences were observed between plants treated with these particles and those not treated, specifically under the RM/14.4 and WRB/8.64 lighting conditions (Figure 1; Table 1).

**Figure 2:**
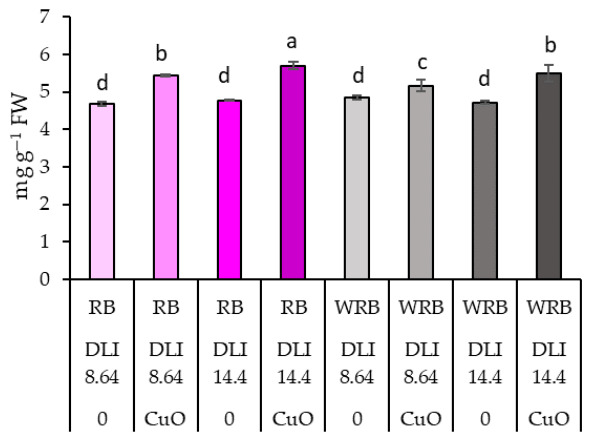
Effects of different lighting and CuO NPs on total proteins. Different letters represent significant differences. RB—red-blue LEDs lamps, WRB—white-red-blue LEDs lamps, DLI—daily light integral, 0—means plants treated with deionised water, CuO—sprayed with CuO NPs, FW—fresh weight. Means with different letters within each column are significantly different at the *p* ≤ 0.05 level according to Tukey’s honestly significant difference test.

Spraying CuO NPs consistently increased total protein content under all illumination conditions, with the most significant increase observed at RM/14.4 (Figure 2; Table 1).

The impact of CuO NPs on the activity of antioxidant enzymes varied significantly under different lighting conditions (Table 2). At a lower DLI (8.64 mol m^−2^ d^−1^), CuO NPs enhanced the activity of nearly all enzymes under both LED spectra used. In contrast, at a higher DLI (14.4 mol m^−2^ d^−1^), these NPs inhibited the activity of the same enzymes, except GR and DHAR, for which no significant differences were observed between lettuce treated with CuO NPs and untreated ones. Notably, SOD demonstrated an increase in activity under the WRB illumination at 14.4 DLI, while its activity decreased under RB illumination at the same DLI under the effect of CuO NPs. When evaluating the effects of individual factors, it was found that, at a higher DLI, WRB lighting increased the activity of antioxidant enzymes, except GR. Generally, CuO NPs stimulated the activity of antioxidant enzymes, but a reliable increase was observed only in DHAR and SOD.

CuO NPs increased DPPH, ABTS, FRAP, and total phenolic compounds under all illumination conditions (Table 3). However, this effect was stronger under WRB and higher DLI illumination conditions. Additionally, CuO NPs enhanced total phenolic compounds specifically under RB LED spectrum conditions.

CuO NPs had no significant impact on carotenoid content (Table 3). However, applying these particles notably increased the xanthophyll content in lettuce leaves. The effect of CuO NPs was more pronounced when lettuce was grown under broad-spectrum LED (WRB) lighting, whereas the daily light integral (DLI) did not show a significant effect.

The effects of the lighting spectrum, DLI, and CuO NPs on the accumulation of mineral elements in lettuce varied by element (Table 4 and Table 5). Notably, these factors did not influence the K content (Table 4). The highest P concentration was observed under RB lighting conditions at a DLI of 8.64 after CuO NPs were applied. However, there were no significant differences in P levels between sprayed and unsprayed plants under either lighting condition. After applying CuO NPs, the highest concentrations of Ca, Mg, and Na were also recorded under RB lighting. Interestingly, a higher DLI led to reduced accumulation of these elements in lettuce. Under WRB LED lighting, contrasting trends in Ca and Mg levels were observed: at a DLI of 8.64, spraying CuO NPs decreased their concentrations, whereas at a DLI of 14.4, it increased them. In WRB lighting, CuO NPs reduced Na content in lettuce under both DLI conditions. Additionally, as DLI increased, S concentration decreased under both lighting spectra. However, the application of CuO NPs did not significantly affect S levels, particularly under WRB LED lighting conditions. After examining the effects of each factor individually, it was observed that the RB LED lighting increased P, S, and Ca levels, whereas the WRB increased Na levels. However, there was no effect on K and Mg. Additionally, the content of S, Ca, Mg, and Na was higher under conditions of lower DLI, while DLI had no effect on P and K. Spraying with CuO NPs did not affect the levels of P, Ca, K, and Mg, but it did reduce the content of S and Na.

CuO NPs significantly enhanced Cu uptake, particularly under broad-spectrum WRB LED lighting and at a higher DLI of 14.4 (Table 5). It was observed that RB LED lighting, regardless of DLI, promoted the accumulation of various microelements, whereas CuO NPs had little effect on their content. The only exceptions were Mn under RB LED lighting at DLI 8.64 and Mo under RB LED lighting at DLI 14.4, where CuO NPs reduced their concentrations. In contrast, WRB LED lighting, combined with a higher DLI of 14.4, significantly enhanced the positive effects of CuO NPs on the accumulation of microelements in lettuce compared with those without these NPs.

**Figure 3:**
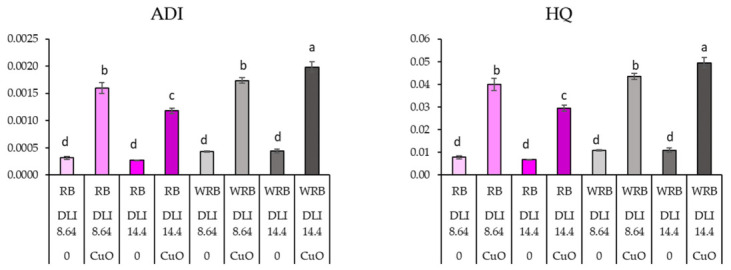
Effects of different lighting conditions and CuO NPs on average daily intake (ADI) and the risk to human health, evaluated using hazard quotients (HQs). RB—red-blue lighting, WRB—white-red-blue lighting, DLI—daily light integral, 0—sprayed with water; CuO—sprayed with CuO NPs. Different letters represent significant differences. Means with different letters within each column are significantly different at the *p* ≤ 0.05 level according to Tukey’s honestly significant difference test.

The studies indicated that higher concentrations of Cu accumulated in plant leaves after spraying with CuO NPs under all lighting conditions. However, the calculated health risk indices showed that the levels of Cu in the edible parts of the plants did not reach toxic thresholds (Figure 3; Table 6). The highest average daily intake (ADI) and hazard quotient (HQ) were observed in lettuce sprayed with CuO NPs and grown under WRM and both 8.64 and 14.4 DLI conditions.

**Figure 4:**
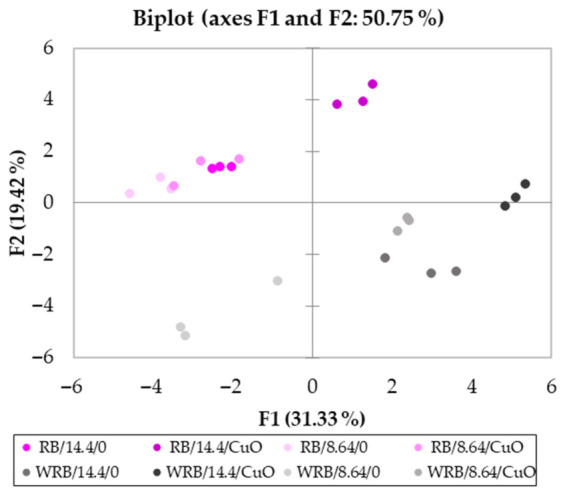
The PCA indicate differences in the investigated parameters of lettuce grown under different lighting and sprayed with CuO NPs. RB—red-blue lighting; WRB—white-red-blue lighting; 8.64 and 14.4—daily light integral; 0—sprayed with water; CuO—sprayed with CuO NPs. The significance level was set at *p* = 0.05.

Principal component analysis (PCA) indicates differences in the investigated parameters of lettuce grown under different lighting conditions and sprayed with CuO NPs (Figure 4). The PCA shows a clear separation of samples along the first principal component (PC1), distinguishing RB samples (negative F1 values) from WRB samples (positive F1 values). This indicates that PC1 captures the primary differences between the two groups. Along the second principal component (PC2), the samples are further separated by treatment conditions, such as DLI and CuO NPs. RB samples generally cluster in the upper-left quadrant, exhibiting positive F2 values, while WRB samples tend to be found in the lower-right quadrant with negative F2 values. This suggests that PC2 represents secondary variations related to the treatment effects rather than the base material itself. Overall, the distinct clustering of samples with minimal overlap between the groups demonstrates strong reproducibility within treatment conditions. It shows that PCA effectively differentiates samples based on both material type and experimental conditions. The first five PCA (F1–F5) had eigenvalues ranging from 9.713 to 2.048 and collectively explained between 31.33% and 87.04% of the variability. F1, which accounted for 31.33% of the total variability, was primarily associated with leaf fresh weight, leaf dry weight, leaf area, xanthophylls, DPPH, ABTS, FRAP, P, S, Ca, Mn, and Mo (Table S2). F2 represented 19.42% of the total variability and included root fresh weight, root dry weight, carotenes, total proteins, total phenolics, B, Na, and Zn. F3 explained 18.48% of the variability and encompassed GR, APX, MDHAR, DHAR, CAT, and SOD. F4 accounted for 11.21% of the variability and included Cu, Fe, K, and Mg. Finally, F5 explained 6.61% of the total variability and was associated with total chlorophylls.

The correlation matrix (Pearson’s) heat map showed light, DLI, CuO NPs and their interaction effect on various lettuce indices (Table S1). Generally, data were analysed when the positive correlation was 0.6 or higher, and the negative correlation was −0.6 or lower. When exploring the effects of individual factors, it was observed that WRB LED lighting showed a strong positive correlation with leaf area, carotenes, DPPH, ABTS, and FRAP, but a strong negative correlation with Mo, B, and Zn compared with RB lighting. DLI 14.4 positively affected plant biomass indices, but negatively affected S, Ca and Mg. Spraying with CuO NPs negatively correlated with total chlorophylls, xantophylls, total proteins and Cu. When exploring the relationship between the LED spectrum and the DLI, we found that fresh and dry leaf weight, leaf area, and the antioxidant activities measured by DPPH, ABTS, and FRAP, as well as Mn levels, showed a positive correlation with WRB × DLI 14.4. Additionally, total phenolic content was positively correlated with RB × DLI 14.4, while P, Ca, and Zn levels were positively correlated with RB*DLI 8.64. There was a strong positive correlation between the WRB relationship with CuO NPs and leaf area, as well as with xanthophylls, DPPH, ABTS, FRAP, and Mn. When analysing the interaction between DLI and CuO NPs, it was observed that DLI 8.64×CuO had the most notable effects, exhibiting moderate negative correlations with indices of growth and antioxidant potential. Additionally, DLI 14.4 × 0 showed a positive correlation with leaf and root fresh and dry weights, total proteins, and Mn. The analysis of the interaction between LED spectrum, DLI, and CuO NPs reveals moderate positive or negative correlations with the examined parameters. Notably, the combination of WRB, DLI of 14.4, and CuO showed moderate to strong positive correlations with several indices, including fresh and dry leaf weight, leaf area, SOD levels, antioxidant activity, and Cu, Fe, and Mn.

The correlation analysis showed that leaf fresh weight, leaf dry weight, root fresh weight, root dry weight, and leaf area are strongly and positively correlated with one another. Additionally, total chlorophyll, xanthophylls, and total proteins are closely intercorrelated. The antioxidant enzymes GR, APX, MDHAR, DHAR, CAT, and SOD form a distinct cluster that is positively correlated, suggesting a coordinated antioxidant defence system in which the activation of one enzyme is associated with the activation of others. Furthermore, the DPPH, ABTS, and FRAP assays are strongly and positively correlated with each other and with leaf area and xanthophylls. However, they are negatively correlated with most mineral nutrients, particularly Zn, which showed a significant negative correlation. The correlations among mineral nutrients varied; strong positive correlations were found between Mo and S and Zn, between Mg and Ca and K, and between Na and Mg, but a negative correlation was observed with B.

## 3. Discussion

The present study demonstrates that spraying with CuO NPs can positively influence lettuce growth. The literature indicates both positive and negative effects of CuO NPs on plant productivity parameters. Some authors observed a significant increase in DW and leaf number in lettuce exposed to CuO NPs via foliar spray compared with the control [10,18,19]. Other authors found no significant impact of spraying CuO NPs on lettuce yield [9,10]. In our studies, we observed a positive effect of CuO NPs exposure from spraying a 30 ppm solution. Hafeez and co-authors [20] found that this concentration doubled wheat leaf area, whereas higher concentrations may cause phytotoxic effects. Xiong and co-authors [12,13] found that higher concentrations of CuO NPs (100–1000 mg L^−1^) reduced lettuce weight, leaf area, and water content, and increased leaf necrosis. However, there is limited information on how CuO NPs spraying effects vary across different lighting conditions. Our data indicated that under the WRB LED lighting with a DLI of 14.4, spraying CuO NPs was more effective at promoting shoot growth than under RB LED lighting. However, both the DLI and CuO NPs had a greater influence on root growth than the light spectrum alone. The literature suggests that a broader lighting spectrum, achieved by combining white LEDs with other spectra, promotes better lettuce growth than RB LED lighting [21,22]. Additionally, our data indicates that spraying CuO NPs enhances this effect. Other research has also indicated that the light spectrum influences the effects of NPs on plant growth and productivity. Research on ice plants showed that applying CuO NPs increased shoot and root weight when grown under HPS lamps, which emit a lower proportion of blue light than LED lamps. Despite this, the overall plant size was greater under LED lighting. This suggests that the plants may have experienced stress under HPS lighting conditions, and that spraying with these NPs could help alleviate that stress [14]. Other studies on NPs and the light spectrum were conducted in vitro. Zia and co-authors [8] stated that the presence of CuO NPs in the media significantly increases the fresh and dry weight of Brassica nigra in vitro grown under green, yellow, and blue lights, depending on the CuO NPs concentration. A similar effect was observed in the same plant when ZnO NPs were added to the media [23]. A recent study showed that the combination of Se NPs and blue LED light significantly enhanced callus growth. This increase was observed in fresh and dry mass, as well as in the number of shoot branches per callus, compared with fluorescent lamps and monochromatic green and red LED lighting [24]. Meanwhile, there is no literature on the combined effects of CuO NPs and light intensity or daily light integrals on plants. However, according to the authors’ data, TiO_2_ NPs enhanced biomass distribution in radish underground parts more significantly when exposed to a higher PPFD of 600 μmol m^−2^ s^−1^ [17]. In contrast, in vitro studies showed that low light intensity (40 μmol m^−2^ s^−1^) and Ag NPs stimulated shoot proliferation in *Phalaenopsis amabilis*, resulting in the highest fresh and dry weights [25].

Chlorophyll is the primary pigment involved in photosynthesis, and variations in its levels significantly affect both photosynthesis and the yield of crops [26]. Our studies indicate that spraying CuO NPs increases chlorophyll content in lettuce under both lighting conditions. The literature indicates that increases in chlorophyll levels may be due to the protective effects of metallic NPs, potentially mediated by antioxidant agents in the chloroplast membrane. The response varies with the dosage used, as CuO NPs can cause adverse effects at certain concentrations and in specific plant species, including lettuce. In contrast, they may have a positive effect or no effect at all in other cases [9,10,19,26,27,28]. We did not find any studies demonstrating the effect of CuO NPs on chlorophyll content in plants under various lighting conditions. However, studies with Ag/ZnO NPs showed that full sun spectrum and red LED light enhanced the positive effects of these NPs on chlorophyll content in wheat seedlings compared to natural sun, blue and blue-red LED light [29].

This study demonstrates that spraying CuO NPs significantly impacts the antioxidant system of lettuce, influencing both enzymatic and non-enzymatic components. Cu is known to generate reactive oxygen species (ROS) in cells through the Fenton reaction. To counteract ROS stress, plants employ both ROS-sequestering enzymes and antioxidant molecules as part of their defence mechanism. Depending on environmental conditions and exposure intensity, CuO NPs can either mildly stimulate defence metabolism or act as phytotoxic agents [9,10,13,27]. Our results indicated that spraying CuO NPs significantly affected the non-enzymatic antioxidant system in lettuce. We observed increases in total antioxidant capacity, as measured by DPPH, ABTS, and FRAP assays, and in phenolic and carotenoid content. This suggests that lettuce enhanced its ability to neutralise free radicals and reduce oxidative intermediates. The observed increase suggests that the concentration of CuO NPs used likely induced a mild oxidative signal, thereby stimulating the plant’s defensive metabolism. The increase in antioxidant activity was more significant under WRB LED lighting and higher DLI. This suggests that using a broader spectrum or more intense light enhances the metabolic response triggered by CuO NPs. Such lighting likely promotes a more balanced excitation of photosystems, thereby improving photosynthetic efficiency and, in turn, increasing carbon availability for the synthesis of secondary metabolites [30]. Other researchers found that lighting influenced the effects of CuO NP spraying on plants. In vitro studies with *Brassica nigra* demonstrated that CuO NPs exhibit varying toxicity across different light wavelengths [8]. Sutulienė and co-authors [31] found that spraying CuO NPs on ice plants did not significantly affect their antioxidant systems under HPS lighting compared to unsprayed plants. However, under LED lighting, there was a notable activation of the antioxidant system, both non-enzymatic and enzymatic. This study on lettuce indicated that spraying with CuO NPs was less effective in stimulating the enzymatic antioxidant system. The most released enzyme was SOD, whose activity increased under WRB LED lighting, particularly at higher DLI levels. It is known that SOD is a crucial enzyme that helps reduce oxidative stress by catalysing the dismutation of superoxide radicals (O2•−). This process reduces the risk of hydroxyl radical (OH•) formation during metal-catalysed reactions. Cu is one of the metal cofactors associated with SOD [32]. Other studies also indicate that the interaction of lighting and NPs influences SOD activity. For example, wheat seedlings exhibited higher SOD activity under 100% red light with Ag/ZnO NPs compared to conditions with a lower percentage of red light [29]. Meanwhile, in vitro studies with sandalwood callus indicated that SOD activity was higher when exposed to blue light and Se NPs compared to illumination from green, red LED, and white, fluorescent lamps [24]. Farrokhzad and co-authors [25] found that the activity of SOD in *Phalaenopsis amabilis* in vitro was higher at a light intensity of 160 μmol m^−2^ s^−1^ and at a concentration of 40 μM Ag NPs in the medium compared to lower light intensities and concentrations of Ag NPs.

This study showed that foliar application of CuO NPs influences the uptake of mineral elements from nutrient solutions. When considering only the impact of CuO NPs, their effect on the accumulation of most macroelements was relatively limited. However, it did lead to a reduction in S and Na levels. In contrast, the microelements content was significantly affected. Foliar spray of CuO NPs induced an increase in the content of B, Fe, Zn, and decreased Mn and had no impact on Mo. Data in the literature on the effects of CuO NPs on levels of other mineral elements are inconsistent. Some authors found that after spraying lettuce leaves with CuO NPs, the concentrations of most macronutrients were higher than in control samples [18,19]. Pérez-Labrada and co-authors [33] stated that applying CuO NPs to tomato leaves increased levels of P, Ca, and Mn, while reducing levels of Na, Fe, and Zn. It was reported that a higher concentration of CuO NPs impaired the uptake of essential elements to varying degrees, including inhibiting the content of Mn, K, and Ca in plant leaves [13]. Studies have demonstrated that, depending on their size, CuO NPs can penetrate through cuticular and non-stomatal pathways after spraying. Therefore, after exposure to leaves, the amount of Cu in the roots also increases, thereby disrupting the absorption of other minerals from the solution. Although dose-dependent (hormetic) reactions have been observed, low concentrations of CuO NPs can stimulate root growth and nutrient uptake [34,35]. Our studies indicated that the concentration of CuO NPs selected for spraying in previous studies did not negatively affect the absorption of mineral elements from the solution, although it significantly increased the Cu content in lettuce leaves. Additionally, lighting had a considerable impact on this matter. The highest Cu concentration was found under WRB LED lighting at a higher DLI of 14.4. Applying CuO NPs under this lighting condition significantly increased the levels of other trace elements compared to other lighting conditions, with almost no significant difference between sprayed and unsprayed plants. However, it was generally observed that the concentrations of various elements increased under RM LED lighting and lower daily light integrals when each factor was considered separately. We did not find any other studies on the combined effects of CuO NPs, LED lighting spectrum, and DLI. However, research on ice plants indicated that high-pressure sodium (HPS) lamp lighting had a greater positive impact on Cu accumulation when plants were sprayed with 30 ppm CuO NPs than LED lighting did. However, there was no notable difference between the sprayed and unsprayed plants under different lighting conditions, except for B, which was significantly reduced in the plants that received the CuO NP spray under HPS lighting [14]. Summarising our results, it can be assumed that under lighting conditions which decrease the absorption of mineral elements, particularly microelements, spraying CuO NPs can enhance this process in lettuce.

This study aimed to investigate not only the effects of CuO NPs foliar sprays on the growth and nutritional value of lettuces but also to assess the accumulation of this metal in edible parts of the plant and the associated potential risks to human health. However, there is relatively limited scientific information on the combined effects of metal NPs and lighting, including the risk of Cu bioaccumulation to human health. It is known that free Cu ions can be toxic to the human body by generating free radicals, and cytotoxic effects on liver and lung cells have also been reported [12,36,37]. In our study, the maximum ADI and HQ values after spraying CuO NPs were highest at WRB with a DLI of 14.4; these values were less than one, indicating they did not exceed the permissible limit. According to the literature, these indicators are exceeded when plants are treated with high concentrations of CuO NPs. However, results indicate that using lower concentrations limits the risk of Cu overdose in the human body, even when plants are grown under lighting that significantly increases Cu content after CuO NPs are sprayed [12,14].

When evaluating the overall effects of CuO NPs on both plants and humans, it is crucial to consider the potential for ion diffusion. The physicochemical properties of the surrounding medium significantly influence the dissolution behaviour of CuO NPs. CuO NPs suspended in ultrapure water show minimal ion release, as the lack of organic ligands and the stable neutral pH reduce the thermodynamic drive for dissolution [38,39]. In low-ionic-strength media, CuO NPs tend to maintain their particulate form, with only a small fraction of the total copper mass transforming into free Cu^2+^ ions. Therefore, in our study, which utilised an ultrapure water suspension for foliar application, it is highly likely that the plants were mainly exposed to CuO nanoparticles rather than ionic copper [38,39]. This suggests that the observed physiological changes are likely due to particle-specific interactions or localised, slow-release kinetics at the leaf surface.

## 4. Materials and Methods

### 4.1. Nanoparticles Preparation and Characteristics

The commercial copper oxide (CuO) nanoparticles (NPs) used for plant exposure in this study were purchased from US Research Nanomaterials (Inc., Houston, TX, USA). Suspensions of CuO NPs (Size: 40 nm, 99% purity) at 30 ppm were prepared in deionised water. The suspensions were prepared in 300 mL flasks by weighing CuO Nps powders, respectively, which were weighed using a highly sensitive balance (Radwag AS 220 R2 PLUS, RADWAG Balances and Scales, Torunska, Poland) and an antistatic ioniser (DJ-04 Antistatic Ioniser, RADWAG Balances and Scales, Torunska, Poland) to remove the static charge of the powder particles. The flasks with suspensions were placed in an ultrasonic bath (Sonerex super ultrasonic bath 80W, Weidinger GmbH, Gernlinden, Germany) and suspended for 60 min. Immediately afterwards, the NPs’ size and suspension stability were measured using a Delsa™Nano Submicron Particle Size (Beckman Coulter Instruments Corporation, Fullerton, CA, USA) and a Zeta Potential device (Dispersion Technology Inc., Bedford Hills, New York, NY, USA). Table 1 shows the positive particle surface charge of the CuO NPs suspensions. The suspensions were stable, according to the zeta potential. In addition, the polydispersity index (PDI) showed that the NPs suspensions were monodisperse.

Plants were sprayed immediately after the ultrasonic bath using automatic sprayers (Rechargeable electric sprayer,1 L, 3.6V, Nozzle hole diameter: 13 mm, Yato, Haiyan, Jiaxing, Zhejiang, China) to the full surface maturity during the first half of the day (15 mL per plant). Before spraying, the plant systems were covered with a plastic sheet to protect the hydroponic solution and roots from exposure to NPs. It should be noted that the persons who carried out the spraying followed all safety requirements, wearing a full protective suit, gloves, and a respirator (Table 7).

### 4.2. Plant Growth Conditions, Lighting, and Nanoparticle Treatments

Experiments were conducted at the Institute of Horticulture in the Lithuanian Research Centre for Agriculture and Forestry. The study was performed in a controlled environment plant growth chamber measuring 4 m by 6 m with a height of 3.2 m. Seeds of the lettuce (*Lactuca sativa*, ‘Little Gem’) were obtained from CN Seeds, Ely, UK. The 200 rockwool cubes measuring 2.5 cm × 2.5 cm × 3.0 cm were used as the growing medium. Before use, the rockwool cubes were soaked in deionised water with a pH of 5.0 adjusted with sulfuric acid, then placed in a plastic tray. Germinated seedlings were grown in this setup for 29 days at a day/night temperature of 21 ± 1/17 ± 1 °C and humidity of 60 ± 5%. The germinated plants were watered with enough hydroponic solution to cover the bottom of the rockwool cubes by 1 centimetre daily. Then, they were transferred to Ebb-type hydroponic systems with 80 L containers containing a hydroponic solution made of deionised water and nutrients in the following quantities (mg L^−1^): N (120), Ca (88), P (20), K (128), Mg (40), S (53), B (0.16), Mo (0.2), Mn (0.08), Cu (0.08), Fe (1.6), Zn (0.8). The electrical conductivity (EC) of the nutrient solution was 1.4 mS cm^−1^, and pH was measured daily with a portable metre (GroLine HI9814, Hanna Instruments, Woonsocket, RI, USA) and adjusted to 6.0 with sulfuric acid or sodium bicarbonate. During the experiment, the lettuces, as well as their seedlings, were exposed to two different lighting conditions: a combination of blue—455 nm and red—660 nm and a combination of white—380–760 nm (4000K), blue—455 nm, and red—660 nm light-emitting diodes (LEDs, OSRAM Oslon SSL, Ecolight, Vilnius, Lithuania) at a photosynthetic photon flux density (PPFD) of 150 and 250 ± 5 μmol m^−2^ s^−1^ with a 16 h photoperiod (Table 8). The technical specification of the LED is included in the Supplementary Materials, Table S3. PPFD was measured at the plant growth level using a portable spectroradiometer (WaveGo, Wave Illumination, Oxford, Oxfordshire, UK). Based on it and the 16h photoperiod, the daylight integral (DLI) was calculated according to the formula:

DLI (mol m^−2^ d^−1^) = 3.6 × 10^−3^ × PPFD (μmol m^−2^ s^−1^) × photoperiod,h (1)

The lamps were hung separately for each system at a height of about 80 cm above the plant canopy, about 50 cm from the wall and about 150 cm between the systems. This distance was enough to prevent the lighting from overlapping according to the measurements of the spectroradiometer. In total, there were 8 systems in the growth chamber (arranged in tiers) with a separate hydroponic solution reservoir, and 36 plants were grown in each system. The systems with plant cultivation were arranged in a completely randomised design. We had two replicates of each lighting used during one cultivation.

Five days before the end of the experiment, lettuces were sprayed with CuO NPs suspensions (preparation described in Section 4.1). Then, the plant growth parameters were measured from 9 plants, and material from 5 plants from each system was used for enzyme analyses, and the remaining plants were lyophilised for other analyses.

### 4.3. Growth Characteristics

The growth characteristics of lettuce were studied by measuring the fresh (FW) and dry weights (DW) of shoots and roots, and leaf area. The FW was measured using an electronic scale (Mettler Toledo, ML104T/00; Mettler-Toledo, Columbus, OH, USA). The DW was determined by drying the divided shoots and roots samples for 48 h in a drying oven (Venticell-222, Medcenter Einrichtungen, Gräfeling, Germany) at 70 °C. The leaf area was measured using a leaf area metre (CI-202 Laser Area Meter; CID BioScience, Camas, WA, USA).

### 4.4. Biochemical Analysis

All biochemical analyses were performed using a spectrometer-based absorbance microplate reader SPECTROstar Nano (BMG Labtech microplate reader, Ortenberg, Germany). Analyses were performed using the flat-bottom, polystyrene, pure-grade, transparent 96-well microplates of 350 μL volume (Brand GmbH + CO KG, Wertheim, Germany) and using the flat-bottom, transparent, cyclic olefin copolymer 96-well microplates of 340 μL volume with 230 nm detection limits (Greiner Bio-One GmbH, Kremsmünster, Austria) for antioxidants’ enzymatic activity. Extracts, reaction mixtures, and substrates were added using a 12-channel pipette, selecting pipettes and tips with volumes of 0.5—10 µL, 10—100 µL, and 30—300 µL (Eppendorf Research Plus, Eppendorf North America, Enfield, CT, USA) according to the required volume.

#### 4.4.1. Enzymatic Antioxidants

The extracts used to determine the enzymatic antioxidant activity in lettuce leaves were prepared by grinding 0.5 g of fresh sample with liquid nitrogen and diluting it in 5 mL of extraction buffer (100 mM potassium phosphate buffer, pH 7.8, containing 0.1 mM EDTA). After centrifugation for 10 min at 3000 rpm (Hermle Z300K, Baden-Württemberg, Germany), the supernatant was collected for enzymatic activity assays. All steps in preparing the enzyme extract were carried out at 4 °C.

Monodehydroascorbate reductase (MDHAR, E.C. 1.6.5.4) activity was assayed by recording the decrease in optical density at 340 nm due to ascorbic acid, as described by Hossain [40], with modifications adapted for a microplate reader by Murshed [41]. The 185 μL assay mixture contained 0.1 M potassium phosphate buffer (pH 7.8, containing 0.1 mM EDTA), 2.5 mM ascorbic acid (AsA), 0.25 NADH, 10 μL enzyme extract, and 5 μL of 80 units mL^−1^ ascorbate oxidase (AsO, Merck KGaA, Darmstadt, Germany), which was added to initiate the reaction. The decrease in absorbance was measured spectrophotometrically for 5 min. The extinction coefficient of 6.22 mM^−1^ cm^−1^ was used to calculate enzyme activity, which was expressed as units AsO min^−1^ mg^−1^ protein.

Dehydroascorbate reductase (DHAR, M.C.1.8.5.1) activity was assayed by measuring the decrease in optical density at 265 nm due to ascorbic acid, as described by Nakano and Asada [42], with modifications adapted for a microplate reader by Murshed [41]. The 185 μL assay mixture contained 0.1 M potassium phosphate buffer (pH 7.8, containing 0.1 mM EDTA), 2.5 mM glutathione (GSH), 10 μL enzyme extract, and 5 μL of 8 mM dehydroascorbate (DHA, Merck KGaA, Darmstadt, Germany), which was added to initiate the reaction. The decrease in absorbance was measured spectrophotometrically for 5 min. The extinction coefficient of 14 mM^−1^ cm^−1^ was used to calculate enzyme activity, expressed as nmol DHA mg^−1^ protein min^−1^.

Glutathione reductase (GR, E.C. 1.6.4.2) activity was measured by monitoring the decrease in absorbance of oxidised glutathione (GSSG) at 340 nm, as described by Smith [43], with modifications adapted for a microplate reader by Murshed [41]. The 185 μL reaction mixture contained 0.1 M potassium phosphate buffer (pH 7.8, containing 0.1 mM EDTA), 0.1 mM NADPH, 10 µL enzyme extract, and 5 µL 20 mM GSSG (Merck KGaA, Darmstadt, Germany), and it was added last to initiate the reaction. The decrease in absorbance was measured by a spectrophotometer for 5 min. An absorption coefficient of 6.22 mM^−1^ cm^−1^ was used for calculations. GR activity was defined as nmol NADPH min^−1^ mg^−1^ protein.

Ascorbate peroxidase (APX, E.C. 1.11.1.1) activity was assayed by recording the decrease in optical density at 290 nm due to ascorbic acid, as described by Nakano and Asada [42], with modifications adapted to a microplate reader by Murshed [41]. The 185 μL assay mixture contained 0.1 M potassium phosphate buffer (pH 7.8, containing 0.1 mM EDTA), 0.5 mM AsA, 10 μL enzyme extract, and 5 μL of 30 mM H_2_O_2_ (30%, Merck KGaA, Darmstadt, Germany), which was added to initiate the reaction. The decrease in absorbance was measured spectrophotometrically for 5 min. The extinction coefficient of 2.8 mM^−1^ cm^−1^ for reduced ascorbate was used to calculate enzyme activity, expressed as µmol AsA min^−1^ mg^−1^ protein.

Superoxide dismutase (SOD, E.C. 1.15.1.1) activity was estimated by measuring the inhibition of the photochemical reduction in nitroblue tetrazolium (NBT) by the enzyme, as described by Giannopolitis and Ries [44], with modifications by Dhindsa [45], and adapted to a microplate reader. In total, 120 μL of reaction mixture consisting of 13 mM methionine, 75 µM NBT, 100 mM potassium phosphate buffer (pH 7.8, containing 0.1 mM EDTA), 25 µL enzyme extract, and 25 µL of 2 mM riboflavin was added to initiate the reaction. The tubes were kept at 150 µmol m^−2^ s^−1^ for 1 min to initiate the reaction, then covered. The absorbance was measured after 30 min with a spectrophotometer at 560 nm, and one unit of enzyme activity was defined as the amount of enzyme that reduced the absorbance to 50% compared to tubes lacking enzyme, expressed as unit min^−1^ mg^−1^ protein.

Catalase (CAT, E.C. 1.11.1.6) activity was measured as the disappearance of H_2_O_2_, as described by Aebi [46] with modifications adapted for the microplate reader. First, 10 µL enzyme extract was added to 185 μL of 0.1 M phosphate buffer (pH 7.8, containing 0.1 mM EDTA). The reaction was started by adding 5 µL of 30 mM H_2_O_2_ (30%, Merck KGaA, Darmstadt, Germany). A spectrophotometer measured the decrease in absorbance at 240 nm for 5 min. Enzyme activity was computed using the extinction coefficient of 6.22 mM^−1^ cm^−1^ and expressed as mmol H_2_O_2_ min^−1^ mg^−1^ protein.

#### 4.4.2. Total Protein

The extracts used to determine the soluble protein content in lettuce leaves were prepared by grinding 0.5 g of fresh sample with liquid nitrogen and diluting it in 5 mL of 100 mM potassium-phosphate buffer (pH 7.8, 0.1 mM EDTA). After centrifugation for 10 min at 3000 rpm (Hermle Z300K, Baden-Württemberg, Germany), the supernatant was collected and used for total soluble protein measurement. All the extract’s preparation steps were carried out at 4 °C. The dye-binding method and bovine serum albumin were used as a standard for soluble protein determination. A volume of 20 µL of enzyme extract was mixed with 280 µL of Bradford reagent diluted by 1:5 with DI water. Absorbance was measured after 2 min at 595 nm using a spectrophotometer, as described by Bradford [47] and adapted for the microplate reader.

#### 4.4.3. Non-Enzymatic Antioxidants

The fresh plant material was immediately frozen in liquid N_2_ and lyophilised for antioxidant activity and phenolic compound analysis. Extracts were prepared by mixing 50 mg of lyophilised plant material with 5 mL of 80% methanol (Sigma-Aldrich, St. Louis, MO, USA) and transferring the mixture to a 5 mL polypropylene conical centrifuge tube (Labbox Labware S.L., Barcelona, Spain). After 24 h, the samples were centrifuged for 10 min at 3000 rpm (Hermle Z300K, Baden-Württemberg, Germany), the extracts were filtered through cellulose filters, and the supernatant was used for further analyses. Each of the three biological replicates consisted of at least eight conjugated plants and was repeated in three analytical replicates.

Antioxidant properties of lettuce leaves were evaluated as the DPPH (2-diphenyl-1-picrylhydrazyl), ABTS (2,20-azino-bis (3-ethylbenzothiazoline-6-sulphonic acid)) diammonium salt radical scavenging activities, and Fe^2+^ reducing antioxidant power assay (FRAP); also, total contents of phenolic compounds were determined.

For the DPPH assay [48], a 126.8 μM DPPH (100% purity; Sigma-Aldrich, Burlington, MA, USA) solution in methanol was prepared. Subsequently, 290 μL of the DPPH solution was transferred to a test tube and mixed with 20 μL of the lettuce leaf extract. The absorbance was scanned at 515 nm while reacting for 16 min. The free radical scavenging capacity was expressed as μmol of DPPH radicals scavenged per 1 g of Dry weight (µmol g^−1^ DW). A calibration curve was determined using Trolox (6-hydroxy-2,5,7,8-tetramethoxychroman-2-carboxylic acid; 97% purity; Sigma-Aldrich, Burlington, MA, USA) as an external standard over a concentration range of 0.1 to 0.6 mM (R^2^ = 0.99).

The ABTS radical cation was obtained by incubating the 7 mM ABTS stock solution with 2.45 mM potassium persulfate (K_2_S_2_O_8_; 99% purity; Sigma-Aldrich, Burlington, MA, USA) and allowing the mixture to stand in the dark at room temperature for 12–16 h before use [49]. Thereafter, 20 μL of the prepared sample was mixed with 290 μL of ABTS solution (ABTS stock solution was diluted 1:7), and the absorbance was measured after 11 min (plateau phase) at 734 nm. The ABTS scavenging activity of lettuce leaf extracts was calculated as the difference between the initial absorbance and the absorbance after 10 min of reaction. A calibration curve was determined using Trolox (6-hydroxy-2,5,7,8-tetramethoxychroman-2-carboxylic acid; 97% purity; Sigma-Aldrich, Burlington, MA, USA) as an external standard over a concentration range of 0.1 to 0.8 mM (R^2^ = 0.99). It was expressed as ABTS µmol scavenged per 1 g of dry weight (µmol g^−1^ DW).

The FRAP method is based on the reduction in ferric ions (Fe^3+^) to ferrous ions (Fe^2+^). The fresh reaction mixture was prepared by mixing 300 mM acetate buffer, pH 3.6, with 10 mM TPTZ (2,4,6-tripyridyl-s-triazine) solution in 40 mM HCl and 20 mM FeCl_3_ × 6H_2_O at 10:1:1 (*v*/*v*/*v*) [50]. Subsequently, 20 µL of the sample was mixed with 290 μL of the reaction mixture and incubated in the dark for 30 min. Readings at 593 nm of the coloured product of the ferrous tripyridyl-triazine complex were then taken. A calibration curve was determined using Fe_2_(SO_4_)_3_ (iron (III) sulfate; 97% purity; Sigma-Aldrich, Burlington, MA, USA) as an external standard over a concentration range of 0.005 to 0.5 mM (R^2^ = 0.99). The antioxidant power is expressed as Fe^2+^ antioxidant capacity (Fe^2+^ µmol g^−1^ DW).

The total content of phenolic compounds was determined as gallic acid equivalents. A 20 µL aliquot of the sample extract was mixed with 20 µL of 10% (*w*/*v*) Folin–Ciocalteu reagent and 160 µL of 1 M Na_2_CO_3_ solution [51]. After incubation for 20 min in the dark, the absorbance was measured at 765 nm. The total quantity of phenolic compounds in mg g^−1^ was calculated from the calibration curve of gallic acid (0.01–0.1 mg mL^−1^, R^2^ = 0.99).

For the determination of photosynthetic pigment content, about 0.05 g of dry, ground plant material was diluted with 3 mL of 80% acetone. The extraction was carried out for 24 h at +4 °C temperature. Then, the extract was centrifuged at 10,000 rpm for 10 min and filtered through a 0.22 µm Nylon syringe filter (VWR International, Radnor, PA, USA). Contents of carotenoids were evaluated using a Shimadzu HPLC system (Shimadzu, Japan) consisting of DGU-14A vacuum degasser, LC-10AT HPLC pumps, SIL-20AC autosampler, CTO-10AS column oven, and SPD-M10A diode array detector (DAD). Separation of compounds was performed using isocratic elution with methanol and acetone (1:1) on a YMC C30 carotenoid column (250 × 3 mm, 5 µm) at 25 °C; a flow rate of 0.6 mL/min was used. Quantitation of compounds was performed from the DAD chromatogram obtained at 450 and 650 nm for carotenoids and chlorophylls, respectively. An external calibration standard method was used for quantitation [52].

### 4.5. Elemental Composition of Lettuce

The macro and microelement concentrations in lettuce leaves were determined by microwave digestion followed by inductively coupled plasma optical emission spectrometry. Complete digestion of dry plant material (0.3 g) was achieved with 8 mL 65% HNO_3_ using a microwave digestion system, Multiwave GO (Anton Paar GmbH, Graz, Austria). The digestion programme was as follows: (1) 170 °C reached within 3 min, digested for 10 min; (2) 180 °C reached within 10 min, digested for 10 min. Fully digested samples were diluted to 50 mL with deionised water. The elemental profile was analysed by an ICP–OES spectrometer (Spectro Genesis, SPECTRO Analytical Instruments, Kleve, Germany). The operating conditions employed for ICP-OES determination were 1300 W RF power, 12 L min^−1^ plasma flow, 1 L min^−1^ auxiliary flow, 0.8 L min^−1^ nebuliser flow, and 1 mL min^−1^ sample uptake rate. The analytical wavelengths chosen were 213.618 nm for P, 766.491 nm for K, 279.079 nm for Mg, 589,592 nm for Na, 445.478 nm for Ca, 324.754 nm for Cu, 257.611 nm for Mn, 259.941 nm for Fe, 213.856 nm for Zn, 249.773 nm for B, and 208,414 nm for Mo. The operating conditions employed for the ICP-OES were: 1.3 kW RF power, 1.0 L min^–1^ auxiliary argon (Ar) flow, 0.80 L min^–1^ nebuliser Ar flow, 12 L min ^–1^ coolant Ar flow, and axial plasma configuration. Each sample was analysed in triplicate. The calibration standards were prepared by diluting a stock multi-elemental standard solution (1000 mg L^−1^) in 6.5% (*v*/*v*) nitric acid and by diluting stock phosphorus and sulphur standard solutions (1000 mg L^−1^) in deionised water. The calibration curves for all the studied elements ranged from 0.01 to 400 mg L^−1^. The contents of macro and microelements in the DW of lettuce are presented [53,54].

### 4.6. Intake Risk Assessment

The average daily intake (mg kg^−1^ day^−1^) of potentially toxic metals by consuming leaves of lettuces after foliar application of CuO NPs was calculated by the equation:

(2) $$ADI=\frac{C_{m}\times C_{f}\times IR}{Bw},$$

C_m_—the metal concentration in a plant (mg kg^–1^) on a dry weight basis.

C_f—_is the conversion factor (0.085).

IR—the ingestion rate of vegetables.

Bw—the average body weight for an adult is 70 kg.

The average daily intake of leafy vegetables was estimated at 100 g (0.1 kg person^–1^ day^–1^).

The risk of non-carcinogenic health effects is often evaluated from hazard quotients (HQs), which are the ratio of the daily intake (often the average daily intake, ADI) to a toxicological reference dose (RfD) according to equation [55]:

(3) $$HQ=\frac{ADI}{RfD}$$

RfD—the oral reference dose for CuO is 0.3 mg kg^–1^ day^–1^ [56] and Cu 0.04 mg kg^–1^ day^–1^ [57].

If the value of HQ is less than 1, it is assumed to be safe from the risk of non-carcinogenic effects. Conversely, if the HQ is equal to or greater than 1, it indicates a potential risk that some exposed individuals may experience adverse health effects.

### 4.7. Statistical Analysis

Microsoft Excel 2010 and XLStat 2020 (Addinsoft, Paris, France) were used for data processing. Analysis of variance (ANOVA) was performed, along with the Tukey multiple comparison test (*p* ≤ 0.05).

## 5. Conclusions

The study demonstrated an interactive effect of foliar-applied CuO NPs and lighting conditions on lettuce growth and physiology. CuO NPs positively influenced plant growth and enhanced the antioxidant system, particularly non-enzymatic components such as phenolics, carotenoids and total antioxidant capacity, with higher effects under broad-spectrum white-red-blue light and higher daily light integral (14.4). The application of CuO NPs significantly increased the copper content in lettuce. Importantly, the concentration of CuO NPs used did not pose any risk to human health, as the estimated intake levels remained below safe limits. Overall, this study demonstrated that the interaction between NPs and lighting plays a crucial role in determining plant physiological responses; however, this area is still not sufficiently explored and requires further research.

## Figures and Tables

**Table 1:** The impact and interaction of different lighting and CuO NPs on growth parameters, total chlorophylls and total protein content in lettuce leaves.

	Shoot Fresh Weight, g	Shoot Dry Weight, g	Root Fresh Weight, g	Root Dry Weight, g	Leaf Area, cm^2^	Total Chlorophylls, mg g^−1^ DW	Total Proteins, mg g^−1^ FW
LED	*	*	*	*	*	*	ns
DLI	*	*	*	*	*	ns	*
CuO NPs	*	*	*	*	*	*	*
LED × DLI	*	*	*	*	*	*	*
LED × CuO NPs	*	*	*	*	*	*	*
DLI × CuO NPs	*	*	*	*	*	*	*
LED × DLI × CuO NPs	*	*	*	*	*	*	*

* shows significant differences, ns—no significant differences. Significant at the *p* ≤ 0.05 level.

**Table 2:** Effects of different lighting and CuO NPs on enzymatic antioxidant activity.

Lighting	DLI	NPs	GR, nmol NADPH min^1^ mg^−1^ Protein	APX, mmol H_2_O_2_ min ^−1^ mg^−1^ Protein	MDHAR, Unit AsO min ^−1^ mg^−1^ Protein	DHAR, nmol DHA min^−1^ mg^−1^ Protein	CAT, mmol H_2_O_2_ min^−1^ mg^1^ Protein	SOD, Unit min^−1^ mg^−1^ Protein
RB	8.64	0	516.6 d	3.76 bc	1.36 bc	431.8 c	5.57 b	528.4 e
		CuO	545.1 b	4.81 a	1.51 a	499.7 ab	6.11 a	690.5 c
	14.4	0	585.7 a	5.00 a	1.57 a	490.9 b	6.22 a	703.9 c
		CuO	491.8 e	3.40 c	1.32 c	435.6 c	5.25 c	401.9 f
WRB	8.64	0	462.4 f	3.71 bc	1.31 c	421.7 c	5.41 bc	585.9 d
		CuO	554.8 b	4.96 a	1.55 a	516.31 a	6.32 a	759.6 b
	14.4	0	540.2 bc	5.07 a	1.53 a	500.6 ab	6.25 a	662.7 c
		CuO	522.8 cd	4.10 b	1.42 b	493.6 ab	5.72 b	815.7 a
Significance								
LED			*	*	ns	*	ns	*
DLI			*	ns	ns	*	ns	ns
CuO NPs			ns	ns	ns	*	ns	*
LED × DLI			*	*	ns	*	*	*
LED × CuO NPs			*	*	*	*	*	*
DLI × CuO NPs			*	*	*	*	*	*
LED × DLI × CuO NPs			*	*	*	*	*	*

Notes. RB—red-blue LEDs lamps, WRB—white-red-blue LEDs lamps, DLI—daily light integral, NPs—nanoparticles, 0—means plants treated with deionised water, CuO—sprayed with CuO NPs. Different letters represent significant differences. Means with different letters within each column are significantly different at the *p* ≤ 0.05 level according to Tukey’s honestly significant difference test. * shows significant differences, ns—no significant differences. Significant at the *p* ≤ 0.05 level.

**Table 3:** Effects of different lighting conditions and CuO NPs on non-enzymatic antioxidant activity of lettuce.

Lighting	DLI	NPs	DPPH, mM TE g^−1^ DW	ABTS, mM TE g^−1^ DW	FRAP, µmol g^−1^ DW	TPC, mg g^−1^ DW	Carotenoids, mg g^−1^ DW	Xanthophylls, mg g^−1^ DW
RB	8.64	0	387.6 e	87.9 g	649.2 e	21.58 cd	90.30 a	503.7 d
		CuO	412.2 e	92.9 f	670.7 e	24.30 bc	71.92 b	576.7 bc
	14.4	0	400.3 e	91.7 fg	675.3 e	25.06 b	72.40 b	504.5 d
		CuO	482.0 d	116.0 d	745.2 d	29.04 a	76.08 b	605.5 abc
WRB	8.64	0	511.0 c	98.4 e	860.5 c	15.04 e	93.06 a	499.5 d
		CuO	633.3 a	149.0 a	1052.8 a	25.03 b	89.94 a	652.3 a
	14.4	0	597.0 b	131.9 c	990.2 b	18.82 d	89.72 a	562.8 c
		CuO	654.8 a	141.5 b	1023.3 ab	21.22 d	98.90 a	633.9 ab
Significance								
LED			*	*	*	*	*	*
DLI			*	*	*	*	ns	*
CuO NPs			*	*	*	*	ns	*
LED × DLI			*	*	*	*	*	*
LED × CuO NPs			*	*	*	*	*	*
DLI × CuO NPs			*	*	*	*	ns	*
LED × DLI × CuO NPs			*	*	*	*	*	*

Notes. RB—red-blue LEDs lamps, WRB—white-red-blue LEDs lamps, DLI—daily light integral, NPs—nanoparticles, 0—means plants treated with deionised water, CuO—sprayed with CuO NPs, DW—dry weight. Different letters represent significant differences. Means with different letters within each column are significantly different at the *p* ≤ 0.05 level according to Tukey’s honestly significant difference test. * shows significant differences, ns—no significant differences. Significant at the *p* ≤ 0.05 level.

**Table 4:** Effects of different lighting conditions and CuO NPs on macroelements (mg g^−1^ DW).

Lighting	DLI	NPs	P	Ca	K	Mg	Na	S
RB	8.64	0	0.57 ab	9.66 b	42.29 a	2.66 bc	1.01 cde	0.120 a
		CuO	0.60 a	10.48 a	42.23 a	3.06 a	1.21 b	0.117 ab
	14.4	0	0.56 ab	9.11 bc	41.21 a	2.32 de	0.97 de	0.118 ab
		CuO	0.52 b	8.28 de	40.56 a	2.18 e	0.89 e	0.109 c
WRB	8.64	0	0.51 b	9.58 bc	42.55 a	2.82 ab	1.44 a	0.115 b
		CuO	0.52 b	9.02 bc	41.69 a	2.56 bcd	1.06 cd	0.114 b
	14.4	0	0.52 b	8.24 e	40.69 a	2.45 cde	1.14 bc	0.106 c
		CuO	0.53 b	8.92 cd	41.66 a	2.59 bcd	1.04 cd	0.109 c
Significance								
LED			*	*	ns	ns	*	*
DLI			ns	*	ns	*	*	*
CuO NPs			ns	ns	ns	ns	*	*
LED × DLI			*	*	ns	*	*	*
LED × CuO NPs			*	*	ns	ns	*	*
DLI × CuO NPs			ns	*	ns	*	*	*
LED × DLI × CuO NPs			*	*	ns	*	*	*

Notes. RB—red-blue lighting, WRB—white-red-blue lighting, DLI—daily light integral, NPs—nanoparticles: 0—sprayed with water, CuO—sprayed with CuO NPs, DW—dry weight, P—phosphorus, Ca—calcium, K—potassium, Mg—magnesium, Na—sodium. Different letters represent significant differences. Means with different letters within each column are significantly different at the *p* ≤ 0.05 level according to Tukey’s honestly significant difference test. * shows significant differences, ns—no significant differences. Significant at the *p* ≤ 0.05 level.

**Table 5:** Effects of different lighting and CuO NPs on microelements, μg g^−1^ DW.

Lighting	DLI	NPs	Cu	B	Fe	Mn	Zn	Mo
RB	8.64	0	2.59 d	8.57 ab	64.70 ab	60.01 b	47.88 a	2.39 a
		CuO	13.16 b	8.07 ab	72.20 a	40.69 c	48.20 a	2.20 ab
	14.4	0	2.26 d	7.92 ab	64.55 ab	54.74 b	44.55 b	2.25 ab
		CuO	9.71 c	9.14 a	64.65 ab	59.92 b	46.53 ab	1.75 c
WRB	8.64	0	3.56 d	3.66 d	55.57 b	52.58 b	32.51 c	1.80 c
		CuO	14.31 b	7.61 ab	56.94 b	57.05 b	31.00 c	1.91 bc
	14.4	0	3.61 d	4.15 c	55.40 b	52.32 b	25.25 d	1.28 d
		CuO	16.32 a	7.02 b	72.25 a	111.40 a	32.66 c	1.82 c
Significance								
LED			*	*	*	*	*	*
DLI			ns	ns	ns	*	*	*
CuO NPs			*	*	*	*	*	ns
LED × DLI			*	*	*	*	*	*
LED × CuO NPs			*	*	*	*	*	*
DLI × CuO NPs			*	*	*	*	*	*
LED × DLI × CuO NPs			*	*	*	*	*	*

Notes. RB—red-blue lighting, WRB—white-red-blue lighting, DLI—daily light integral, NPs—nanoparticles: 0—sprayed with water; CuO—sprayed with CuO NPs, DW—dry weight, Cu—copper, B—boron, Fe—iron, Mn—manganese, Zn—zinc, Mo—molybdenum. Different letters represent significant differences. Means with different letters within each column are significantly different at the *p* ≤ 0.05 level according to Tukey’s honestly significant difference test. * shows significant differences, ns—no significant differences. Significant at the *p* ≤ 0.05 level.

**Table 6:** The impact and interaction of different lighting conditions and CuO NPs on average daily intake (ADI) and the risk to human health, evaluated using hazard quotients (HQs) in lettuce.

	ADI	HQ
LED	*	*
DLI	ns	ns
CuO NPs	*	*
LED × DLI	*	*
LED × CuO NPs	*	*
DLI × CuO NPs	*	*
LED × DLI × CuO NPs	*	*

* shows significant differences, ns—no significant differences. Significant at the *p* ≤ 0.05 level.

**Table 7:** Properties of CuO NPs suspensions in deionised water: polydispersity index (PDI) and zeta potential (ZP) results represent the mean ± standard error and percentage of NPs between 1 and 100 nm in the suspension.

	CuO, 40 nm, 30 ppm
PDI	0.267 ± 0.029
ZP (mV)	10.48 ± 1.916
Particle size in suspension up to 100 nm; %	68.2

**Table 8:** Photosynthetic photon flux density.

Lighting			Lighting Code	5000 K	660 nm	455 nm
Spectrum	Intensity, µmol m^−2^ s^−1^	DLI		µmol m^−2^ s^−1^		
R90%:B10%	150	8.64	RB/8.64	0	135 ± 2	15 ± 2
	250	14.4	RB/14.4	0	215 ± 10	35 ± 8
W65%:R30%:B5%	150	8.64	WRB/8.64	98 ± 2	45 ± 2	8 ± 2
	250	14.4	WRB/14.4	155 ± 10	72 ± 3	12 ± 2

## Data Availability

All the data are available upon request.

## Supplementary Materials

The following supporting information can be downloaded at: https://www.mdpi.com/article/10.3390/plants15101477/s1, Table S1: Correlation matrix (Pearson’s) heat map; Table S2. Factor loadings; Table S3. Technical specification of LEDs; Figure S1. Image of the correlation matrix.

## Abbreviations

The following abbreviations are used in this manuscript:

PPFD	PPFD Photosynthetic Photon Flux Density
LED	LED light-emitting diodes
RB	Red-blue LED lighting
WRB	White-red-blue LED lighting
DLI	Daily light integral
FW	Fresh weight
DW	Dry weight
ABTS	ABTS 2,2′-azino-bis(3-ethylbenzothiazoline-6-sulfonic acid)
DPPH	DPPH 2,2-Diphenyl-1-picrylhydrazyl
FRAP	FRAP Ferric reducing antioxidant power
TPC	TPC Total phenols content
SOD	SOD superoxide dismutase
APX	APX ascorbate peroxidase
GR	GR glutathione reductase
ADI	Average daily intake
HQ	Hazard quotients
